# Experimental investigations on physico-mechanical properties of kaolinite clay soil stabilized at optimum silica fume content using clamshell ash and lime

**DOI:** 10.1038/s41598-024-61854-1

**Published:** 2024-05-14

**Authors:** Muhammad Syamsul Imran Zaini, Muzamir Hasan, Sultan Almuaythir, Masayuki Hyodo

**Affiliations:** 1 Faculty of Civil Engineering Technology, Universiti Malaysia Pahang Al-Sultan Abdullah, Lebuh Persiaran Tun Khalil Yaakob, 26300 Kuantan, Pahang Malaysia; 2 Department of Civil Engineering, College of Engineering in Al-Kharj, Prince Sattam bin Abdulaziz University, 11942 Al-Kharj, Saudi Arabia; 3 Graduate School of Science and Technology for Innovation, Yamaguchi University, Ube, Japan

**Keywords:** Clamshell ash, Kaolinite clay, Lime, Morphological microstructure, Shrink-swell, Strength, Civil engineering, Environmental sciences

## Abstract

This investigation examines the effect of clamshell ash (CSA) and lime additives on the physico-mechanical characteristics of kaolinite clay soil stabilized at the optimum silica fume content. Laboratory tests were performed to assess plasticity, shrink-swell characteristics, compaction characteristics, unconfined compressive strength (UCS), shear strength characteristics, mineralogical and morphological microstructure characteristics of stabilized soil specimens. The kaolinite clay soil was stabilized at its optimum silica fume content (6%) to produce the highest strength and was altered with three non-identical proportions of clamshell ash and lime (3%-9%). Cylindrical soil specimens, 76 mm in height and 38 mm in diameter, were moulded and treated for curing periods of 1, 7, 14, and 30 days to examine the strength of the altered soil. The findings revealed that, adding clamshell ash and lime significantly alters the plasticity, shrink-swell, maximum dry unit weights, and optimum moisture contents of the silica fume-stabilized soil. In terms of strength, the beneficial effects of CSA and lime additives were found to be more significant with more extended curing periods. Incremental increases in curing periods resulted in further enhancements in UCS, cohesion, and internal friction angle, indicating continued strength development over time. Microstructural analysis using field emission scanning electron microscopy and X-ray diffraction provided insights into the interparticle bonding mechanisms and microstructural changes induced by the addition of CSA and lime. The emergence of cementitious phases and pozzolanic responses between soil particles and stabilizers contributed to the densification and strengthening of the stabilized soil matrix. The findings of this study provide valuable insights into the potential of clamshell ash and lime additives to enhance the engineering properties of kaolinite clay soil stabilized with silica fume. These results have implications for sustainable soil stabilization practices, offering a promising approach to improve the performance of soils for various engineering applications, including construction and geotechnical projects.

## Introduction

The soil stabilization technique is used in civil engineering to ameliorate the engineering characteristics of problematic and expansive clay soils through physical, chemical, mechanical, biological, or mixed approaches to meet the construction development criteria^1–3^. Various soil stabilizing agents have been utilized in the past few decades to improve the compressibility, strength, and durability of the complex behavior of clay soil, which includes the utilization of traditional (cement and lime) or alternative soil binders (silica fume, fly ash, etc.)^2,3^ or the combinations of both^4–7^. However, due to the negative impact resulting from using cement as a soil stabilizer, the construction industry is now migrating to a better approach of utilizing an alternative soil stabilizer that is more environmentally friendly^8–10^. Nonetheless, there is a need to identify suitable substitute binders that can replace cement^11–13^, thus enhancing the problematic and expansive soil characteristics that would have a beneficial economic impact and be environmentally friendly for the construction application^14–17^.

Soil stabilization is a fundamental aspect of geotechnical engineering aimed at improving the engineering characteristics of soils for various construction applications. Kaolinite, or kaolin (Al_2_SiO_5_(OH)_4_), is a clay mineral characterized by its structure^18–21^. This mineral, commonly found in white coloration, is prevalent in soils created through the chemical weathering of rocks under warm and muggy conditions, such as those in tropical rainforest regions. Kaolinite clay soil, categorized by its low plasticity, low shear strength, and susceptibility to shrink-swell behavior, presents substantial challenges in construction projects^19,22^. In response to these challenges, researchers and engineers have examined various soil stabilization techniques, including incorporating supplementary cementitious materials and chemical stabilizers, to improve clay soils' mechanical properties and performance^23–25^.

Soft clays often have a high liquid limit and plasticity index. This implies they can withstand considerable deformation without cracking or crumbling. However, their great flexibility makes them difficult to work with during construction, necessitating specialized techniques for excavation, support, and stabilization. Silica fume (SF), a byproduct of silicon and ferrosilicon alloy production, has captured attention as an effective additive for soil stabilization owing to its pozzolanic reactivity and ability to improve soil-cementitious binder interactions. Kaolinitic clay soils mixed with 4% SF and lime ensued in a depletion in maximum dry density value, an increment in optimum moisture content value, and improved undrained shears strength^19,26,27^. Soft clays mixed with SF and lime significantly increase workability and strength, reducing soil plasticity^28–30^. Expansive soil blended with various proportions of metakaolin and SF significantly altered soil plasticity and compaction parameters^29,31,32^. Stabilizing expansive clays using SF also resulted in trivial alterations in the stabilized clay soils' plasticity, compaction, and strength^31–34^. Moreover, using SF as a soil stabilizer was validated to be practical in altering the swelling and shrinkage of expansive clay soils exposed to wetting–drying cycles^35–38^. However, while SF alone can enhance certain aspects of soil behavior, its effectiveness may be limited, particularly in attaining optimal strength and durability characteristics in kaolinite clay soil.

In the present-day context, there has been intensifying attraction in assessing the combined effects of SF with other supplementary materials to enhance soil stabilization performance. Clamshell ash (CSA), a waste product generated from seafood processing industries, and lime, a traditional stabilizing agent, have emerged as promising materials for soil stabilization owing to their abundance, cost-effectiveness, and environmental sustainability. Calcium carbonate (CaCO3) is the major element of clamshell ash, accounting for more than 90% of its total composition. This calcium carbonate might be calcite, aragonite, or a combination of the two polymorphs^39^. Clamshell ash is chemically stable and inert in most environments. It does not undergo substantial chemical reactions, making it ideal for a variety of industrial and agricultural applications. However, more research has yet to be performed to explore the effects of combining these materials with SF on the physico-mechanical characteristics of kaolinite clay soil. This investigation bridges the disparity by comprehensively examining the impacts of clamshell ash and lime additives on the physico-mechanical characteristics of kaolinite clay soil stabilized at the optimum silica fume content. Through a sequence of laboratory assessments, including plasticity, shrink-swell characteristics, compaction, unconfined compressive strength (UCS), shear strength, and mineralogical analysis, this research tends to examine the combined effects of these stabilizers on soil behavior and performance. Furthermore, microstructural analysis using field emission scanning electron microscopy (FESEM) and X-ray diffraction (XRD) will provide insights into the mechanisms underlying the interaction between additives and the soil matrix. By examining the impact of clamshell ash, lime, and silica fume on clay stabilization, this research advances sustainable and cost-effective soil stabilization techniques with significant implications for civil engineering and construction endeavors.

## Materials and methods

The materials examined in this study are kaolinite clay soil, silica fume, calcinated clamshell ash (CSA), and laboratory-grade hydrated lime utilized as the subsidiary alteration agent in the stabilization method.

### Materials preparations and properties

**Figure 1:**
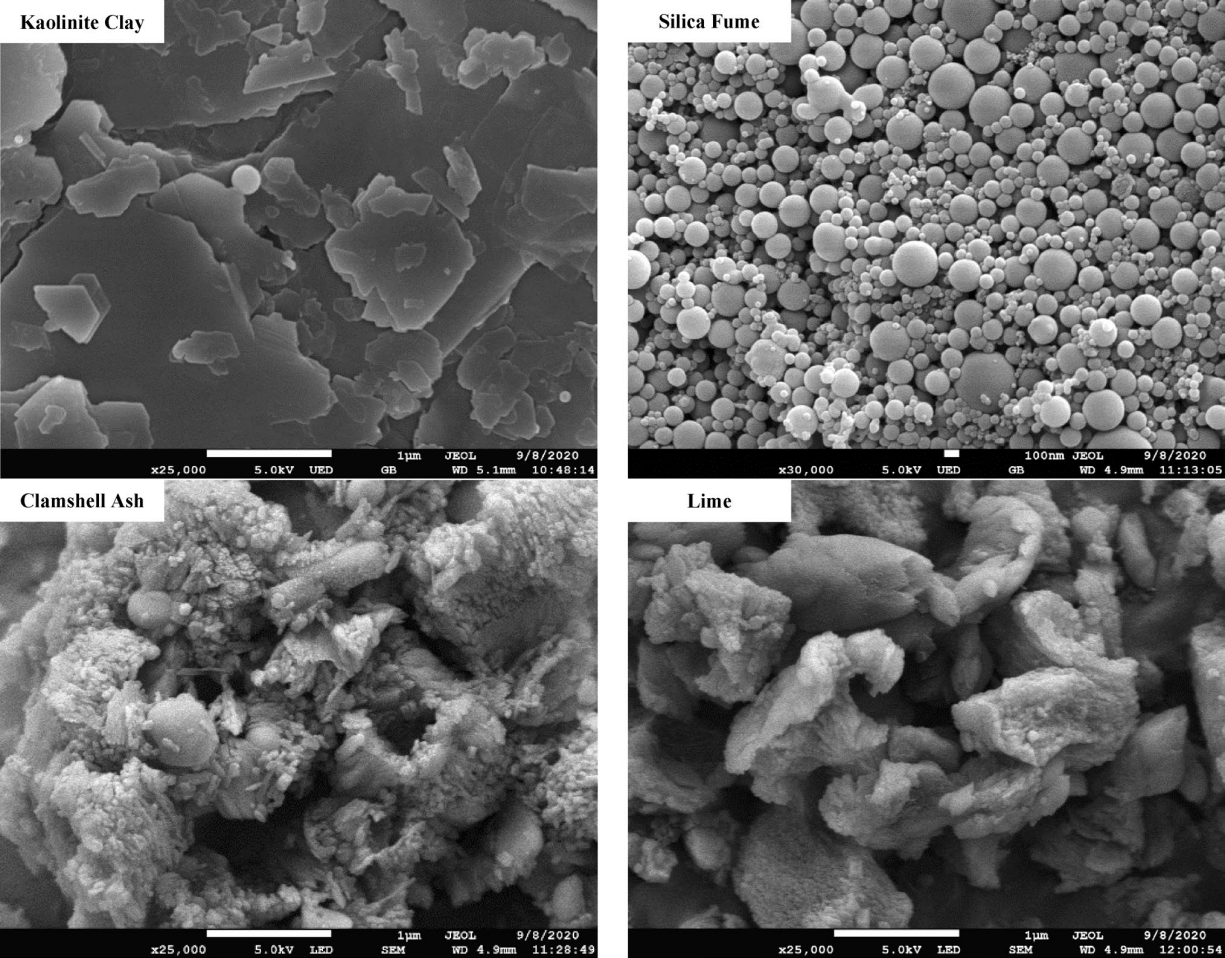
Microstructure of kaolinite clay, SF, CSA, and Lime.

Kaolinite clay is a mineral whose chemical composition is Al_2_Si_2_O_5_(OH)_4_. Kaolinite clay soil has a water-repellent polymer structure and tends to blend with moisture to form uniform clay. The kaolin powder's structure is plate-like, fused by hydrogen bonds and secondary valence forces^19^. The engineering characteristics of the kaolinite soil are listed in Table 1. Silica fume was obtained from Scancem Materials Sdn. Bhd, Malaysia. It is known for its high pozzolanic value owing to its elevated silica content. The clamshells, predominantly composed of CaCO_3_, were cumulated from restaurants in Kuantan, Pahang, and around the east coast beach of Malaysia. These clamshells undergo calcination, resulting in the alteration of CaCO_3_ to CaO. The air-dried clamshells were pulverized using a jaw crusher and then calcined at 800 °C for 1 h. The CSA exhibited a white color from the calcination process. It was subsequently stored in a desiccator for one day, allowing it to cool and preventing the hydration process between CSA and atmospheric moisture. The lime utilized in the study was a laboratory-grade hydrated lime supplied by CAO Industries; it was utilized owing to its compatible chemical compositions, which depleted the probability of variations in laboratory results. The chemical compositions of kaolinite clay, SF, CSA, and lime and their significant mineralogical identifications are listed in Tables 2 and 3. The morphological microstructures of all four (4) materials are illustrated in Fig. 1.

**Table 1:** Geotechnical characteristics of kaolinite clay.

Properties	Unit	Result
Gravel	%	0
Sand	%	40
Clay and Silt	%	60
Moisture Content	%	0.96
Specific Gravity		2.62
Liquid Limit	%	41.1
Plastic Limit	%	33.3
Plasticity Index	%	7.8
Shrinkage Limit	%	23.93
Maximum Dry Density	g/cm^3^	1.55
Optimum Moisture Content	%	21.00
Coefficient of Hydraulic Conductivity	ms^-1^	2.5749 × 10^–8^
Unconfined Compression Strength: 1 Day of Curing 7 Days of Curing 14 Days of Curing 30 Days of Curing	kPa	13.15
Undrained Shear Strength	kPa	6.58
Free Swell Index	%	1.2
pH	–	4.5

**Table 2:** Compositions of untreated soil and soil stabilizers.

Sample type	Compositions (%)						
	SiO_2_	CaO	Al_2_O_3_	K_2_O	MgO	Fe_2_O_3_	Total
Kaolinite Clay	66.11	0.08	20.15	2.85	1.23	0.73	91.15
SF	74.02	0.00	0.55	3.97	3.13	0.69	82.36
CSA	0.92	56.12	1.26	0.00	0.98	0.00	59.28
Lime	1.50	71.90	0.41	0.00	1.13	0.29	75.23

**Table 3:** Mineralogical characteristics of soil, SF, CSA, and Lime.

Properties	Result
Kaolinite Clay	Quartz, kaolinite, and illite
SF	Silica
CSA	Calcium hydroxide, calcite and portlandite
Lime	Calcium hydroxide, calcite and portlandite

### Sample preparation

**Figure 2:**
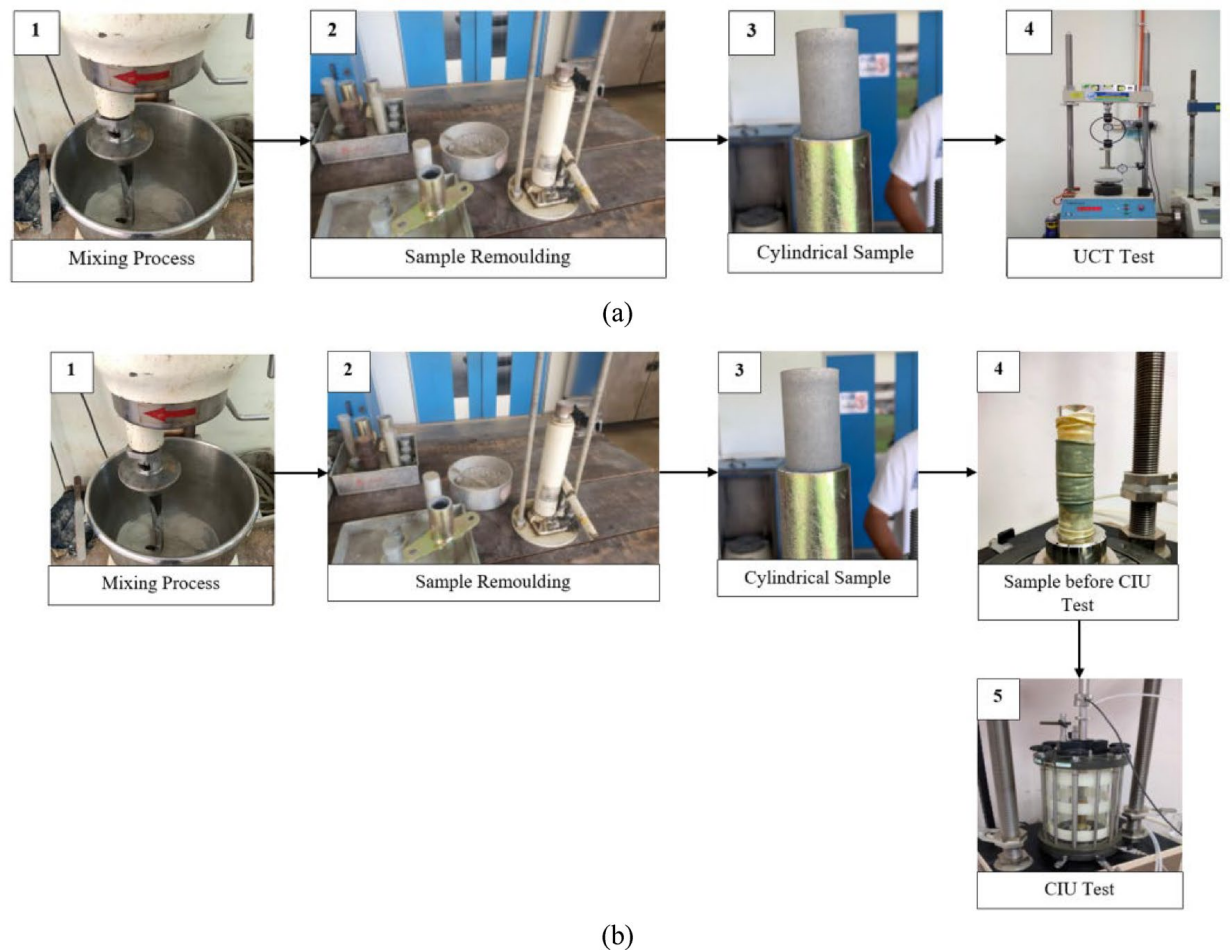
Sample preparation for (a) UCT and (b) CIU Test.

**Figure 3:**
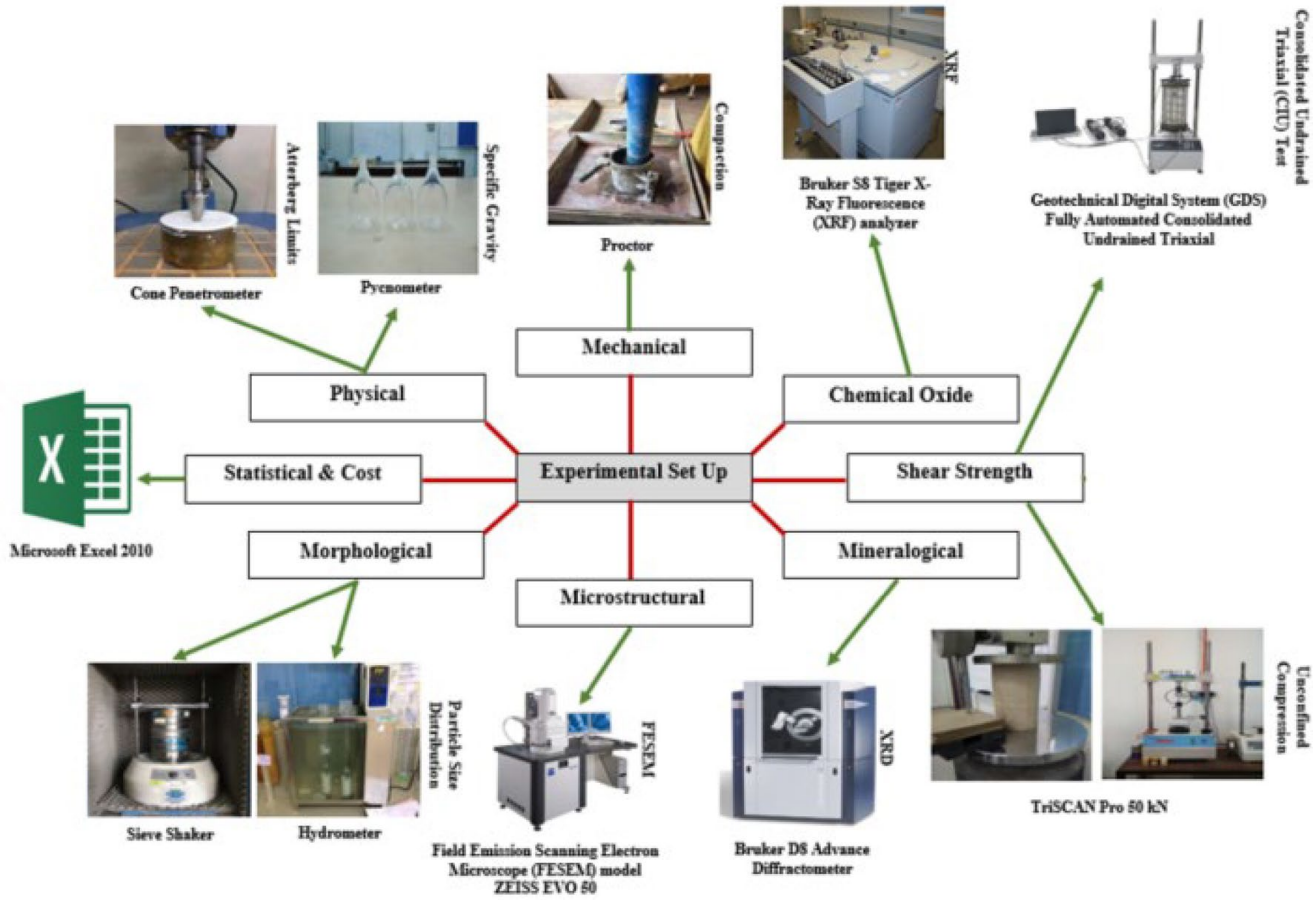
experimental setup of kaolinite soil stabilization.

The optimum silica fume content (OSFC) at 6% of utilization was determined based on the procedure arrogated by past researchers^9,18,19,22^. According to the literature, lime content adopted was selected as 3%, 6%, and 9%^18,26,40^. The CSA content was fixed similarly to the lime to maintain uniformity in investigating and comparing the effectiveness of CSA and lime on the silica fume-stabilized soil (SFSS). In the study, 6% of SF was admixed with 3%, 6%, and 9% of CSA and lime and were tested under eight (8) different tests as highlighted in section "Soil consistency tests" to section "X-ray fluorescence (XRF), X-ray diffraction (XRD), and field emission scanning electron microscopy (FESEM) test". Table 4 shows the sample mixture used in the study and Table 5 shows the sample used coherent to the test standard used in the study. In addition, Fig. 2 portrays the sample preparation for UCT and CIU test, while Fig. 3 portrays the experimental setup of kaolinite soil stabilization implemented in this study.

**Table 4:** Sample mixture.

Type of sample	Percentage of utilization (%)				
	Kaolinite clay	Silica fume	Clamshell ash	Lime	Total
SFSS	94	6	0	0	100
SFSS + 3% CSA	91	6	3	0	100
SFSS + 6% CSA	88	6	6	0	100
SFSS + 9% CSA	85	6	9	0	100
SFSS + 3% Lime	91	6	0	3	100
SFSS + 6% Lime	88	6	0	6	100
SFSS + 9% Lime	85	6	0	9	100

**Table 5:** Sample coding coherent to the test standard utilized in the study.

Type of testing	Standard	Sample used
Soil Consistency Test	BS 1377: Part 2: 1990	SFSS SFSS + 3% CSA SFSS + 6% CSA SFSS + 9% CSA SFSS + 3% Lime SFSS + 6% Lime SFSS + 9% Lime
Swelling Potential Test	ASTM D 4546	SFSS SFSS + 3% CSA SFSS + 6% CSA SFSS + 9% CSA SFSS + 3% Lime SFSS + 6% Lime SFSS + 9% Lime
Compaction Test	BS 1377: Part 2: 1990	SFSS SFSS + 3% CSA SFSS + 6% CSA SFSS + 9% CSA SFSS + 3% Lime SFSS + 6% Lime SFSS + 9% Lime
Unconfined Compression Test	ASTM E1621-16	SFSS SFSS + 3% CSA SFSS + 6% CSA SFSS + 9% CSA SFSS + 3% Lime SFSS + 6% Lime SFSS + 9% Lime
Consolidated Isotropic Undrained (CIU) Triaxial Test	BS 1377: Part 7: 1990	SFSS SFSS + 3% CSA SFSS + 6% CSA SFSS + 9% CSA SFSS + 3% Lime SFSS + 6% Lime SFSS + 9% Lime
XRF	ASTM E1621–13	Kaolinite Clay Silica Fume Clamshell Ash Lime
XRD	ASTM C1365–18	SFSS SFSS + 9% CSA SFSS + 9% Lime
FESEM	N/A	SFSS SFSS + 9% CSA SFSS + 9% Lime

### Soil consistency tests

The plasticity range of clay soil can be measured in numerical expression with consistency limits, as the moisture content of clay soil is also known as plastic consistency. Expansive clay was manufactured using the test method because its particle size range was finer than 63 µm. Clay and soil appear in four (4) states, depending on a specific moisture content: solid, semi-solid, plastic, and liquid. The consistency tests were conducted according to BS 1377: Part 2: 1990. The consistency tests were conducted on air-dried SFSS fraction passing 425 µm sieve. The LL, PL, and SL were based on BS 1377: Part 2: 1990.

### Swelling potential test

A free swell test was performed per ASTM D 4546 to determine the increase in soil volume without applying external pressure by submerging it in water. The test was carried out by passing a 10-g typical oven-dry sample through a 425-micron sieve.

### Compaction test

Standard Proctor compaction test was used in accordance with BS 1377–2:1990 by utilizing a 2.5 kg hammer and 1-L capacity mould to determine the interconnection between the optimum moisture content (OMC) and the maximum dry density (MDD) for treated and untreated soft kaolin clay. Three (3) layers were compacted one by one by dropping the hammer via a free fall method with a distance of about 30 cm from the tip of the hammer to the soil, with a total number of twenty-five (25) blows per layer. The OMC and MDD were determined from the graph plotted between the dry unit weights against the moisture content.

### Unconfined compression test

UCT tests determined the non-dry shear strength of the materials used in this study. The density of treated and non-treated soft clay was 1.74 kg/cm^3^. The density of both samples is uniformly maintained in UCT tests because the data must be maintained for their uniformity. UCT was performed in accordance with ASTM E1621-16 to determine the strength of the soil since the unconfined compression imposed axial load without side restrictions. The samples utilized were prepared by compressing soil specimens with an optimal water content of the soils. The specimens were compressed in a cylindrical steel mould with dimensions of 76 mm height × 38 mm diameter and were treated for 1, 7, 14 and 30 days of curing in air tight container at room temperature of 25 °C. In this test, numerical data on the axial load of the failure and the corresponding axial stress were recorded. A total of five (5) samples were prepared for each of the mixtrue in UCS test.

### Consolidated isotropic undrained (CIU) triaxial test

In this study, the consolidated isotropic undrained (CIU) triaxial test was utilized to evaluate the shear strength of the SFSS and SFSS altered with CSA and lime. The density of treated and non-treated soft clay was 1.74 kg/cm^3^. Eventually, the CIU tests were performed according to BS 1377: Part 7: 1990. The samples utilized in the CIU test were prepared by compressing soil specimens with an optimal water content of the soils. The specimens were compressed in a cylindrical steel mould with dimensions of 76 mm height × 38 mm diameter and were treated for 1, 7, 14 and 30 days of curing in air tight container at room temperature of 25 °C. A total of three (3) samples were prepared for each of the mixtrue in CIU test.

### X-ray fluorescence (XRF), X-ray diffraction (XRD), and field emission scanning electron microscopy (FESEM) test

XRF analysis was conducted utilizing Bruker S8 Tiger X-ray Fluorescence (XRF) analyzer and fully followed the ASTM E1621–13. The Bruker D8 Advance Diffractometer performed the XRD analysis of SFSS and SFSS, which was altered with CSA and lime at position 2-theta (2θ) with compliance to ASTM C1365–18. The FESEM analysis was done on SFSS, and SFSS was altered with CSA and lime in a FESEM model ZEISS EVO 50.

## Results and discussion

The optimum silica fume content (OSFC) for the soil under examination was 6%, which was implemented to stabilize the kaolinite clay soil. The stabilization mechanism was altered by mixing 3%, 6%, and 9% CSA and lime. The influence of the alteration on the plasticity, shrink-swell characteristics, compaction characteristics, unconfined compressive strength (UCS), shear strength parameters, and mineralogical and morphological microstructure properties were examined as part of the study.

### Effect of CSA and lime on the plasticity and shrink-swell properties of SFSS

**Figure 4:**
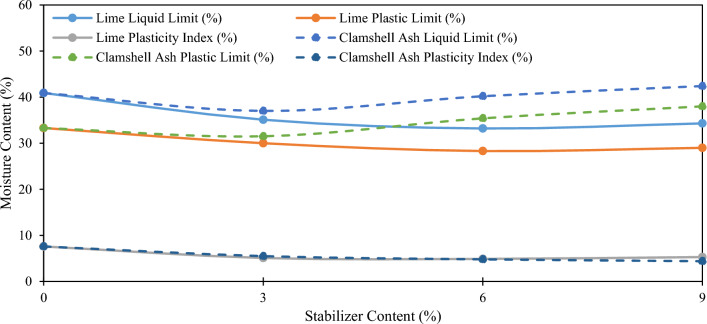
Plasticity of 6% SFSS altered with CSA and lime content.

Figure 4 illustrates the alterations in consistency limits owing to the inclusion of CSA and lime in the stabilized soil with OSFC. The addition of CSA and lime to 6% of SFSS resulted in an initial depletion in the liquid and plastic limits, then increased at further inclusion of CSA and stabilized at further inclusion of lime proportion. The liquid limit and plastic limit diminished to the lowest value of 37.0% and 31.5% at 3% of CSA inclusion, while at 6% of lime inclusion, the liquid limit and plastic limit diminished to the lowest value of 33.2% and 28.3%. Coherent to that, it is evident that at 6% of SF content, CSA and lime modify the plasticity of the SFSS by modifying both the liquid and plastic limits of the stabilized soil. The outcome of the plasticity test discloses that plasticity is the lowest at 3% CSA and 6% lime at 5.5% and 4.9%, respectively, as opposed to a value of 6.6% of pure SFSS. The plasticity modifications of SFSS induced by CSA and lime inclusion were affected by the cation interchange that exists between the SFSS and positive cations in the CSA and lime. In SFSS, the addition of CSA and lime causes cation exchange processes that drastically modify the soil's plasticity. Cation exchange refers to the transfer of positively charged ions (cations) between the SFSS and the additional components. CSA and lime produce calcium (Ca^2+^) ions, which replace other cations found in the soil, including as sodium (Na^+^) and potassium (K^+^). This exchange lowers the concentrations of sodium and potassium ions in the soil, reducing its flexibility. Furthermore, aluminum (Al^3+^) ions from CSA may replace other cations, altering soil characteristics. Lime dissociates into calcium (Ca^2+^) and hydrogen (H^+^) ions, with the hydrogen ions displacing other cations and causing soil flocculation, hence decreasing flexibility. Overall, the cation exchange mechanism involving calcium, aluminum, and hydrogen ions has a significant impact on the flexibility of SFSS when stabilized with CSA and lime, resulting in improved engineering characteristics and stability.

The depletion in plasticity properties of the SFSS was affected by substituting highly plastic SF-clay particles with unamiable CSA and lime molecules. At higher CSA and lime contents, the plasticity increases and becomes stable. This is because, when CSA comes into contact with water, it forms calcium silicate hydrate (CSH) gel and calcium aluminate silicate hydrate (CASH) gel due to its high concentration of calcium oxide (CaO) and lime (calcium hydroxide, Ca(OH)_2_). These hydrated gels cover the gaps between soil particles, increasing cohesion and lowering soil flexibility. Furthermore, the extra lime content produces an excess of calcium ions, which participate in cation exchange activities, replacing exchangeable sodium and potassium ions in the soil. This exchange process further lowers soil plasticity by flocculating clay particles. As the lime level rises, surplus lime combines with soil particles to generate stable calcium silicate and calcium aluminate compounds, increasing soil stability while decreasing flexibility. As a result, at higher CSA and lime contents, the soil's plasticity initially increases due to the presence of excess lime and reactive materials, but then stabilizes as the soil undergoes hydration and chemical reactions, resulting in improved engineering properties and stability^41,42^. Hence, low CSA and lime contents are more efficacious in minimizing plasticity characteristics than higher percentages of CSA and lime with higher SF content. XRF analysis shows that CSA and lime contain substantial amounts of calcium oxide (CaO). The consistency limits results obtained in this study are in a good agreement with an investigation performed by Harichane et al.^12^. The utilization of pozzolanic materials can significantly modify the consistency limits of the clayey soils.

James^43^ and Bian et al.^44^ validated that the inclusion of SiO_2_ and CaO-based materials in SFSS soils causes flocculation, thus reducing the consistency limits of the soil. Moreover, due to the fact that SFSS is alumino-silicates, the response of CaO with the SFSS structure generates CSH and CAH, which equalize the ionic bond in the structure of SFSS minerals and reduce the exchange capacity of the cation for water molecules. According to Mohanty et al.^45^, the depletion in moisture retention capacity can be ascribed to the presence of stabilizing agents that coat the SFSS molecules, causing them to bind together and fill the voids within the soil matrix. This process leads to a decrease in pore space and moisture content. Additionally, the incorporation of clamshell ash (CSA) and lime changes the consistency limits of the soil-stabilizing system (SFSS) due to increased affinity for moisture. CSA and lime facilitate the cohesion and aggregation of SFSS particles, resulting in a coarser texture. Similar findings were reported by Alrubaye et al.^19^ and Zaini and Hasan^26,40,46^.

**Figure 5:**
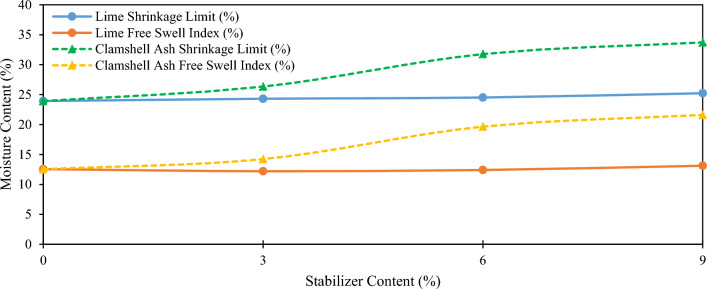
Effect of CSA and lime on the shrink-swell characteristics of 6% SFSS.

As the soil undergoes drying, the molecules comprising the soil-stabilizing system (SFSS) exhibit proximity, leading to a decrease in moisture content. Despite this reduction in moisture, the volume of the SFSS mass remains relatively unchanged, as it assumes a solid-like behavior. This phase, characterized by a stabilized moisture content, is termed the shrinkage limit (SL), which profoundly influences the propensity of the SFSS to undergo shrinkage. Notably, a lower SL value correlates with a heightened capacity for shrinkage within the SFSS. Figure 5 highlights the shrink-swell characteristics of CSA and lime-altered SFSS. The inclusion of CSA and lime resulted in a minimal upsurge in the FSI of the stabilized soil. The FSI of the SFSS raised from 12.55 to 21.61 and 13.12% only, with a margin difference of 9.06% and 0.57% when the maximum proportion of CSA and lime were utilized. The results show that the FSI of the SFSS when CSA was utilized was significantly improved, while opposite results were obtained when lime was integrated into the SFSS. The integration between SFSS molecules with CSA and lime molecules resulted in the massive repository of calcium ions as CSA, and lime has substantial CaO content. The increase in swelling is attributed to the SFSS's elevated capacity for swelling upon exposure to water, stemming from its capability to absorb and retain water molecules. The integration of CSA and lime significantly influences the SL of the SFSS. The difference between the SL value observed in pure SFSS specimens and SFSS altered with CSA and lime exceeds 1%.

Nonetheless, the inclusion of CSA and lime significantly alters the shrinkage characteristics of SFSS to an enormous extent. In line with this, the elevation in shrinkage limit (SL) is ascribed to the aggregation of molecules resulting from clamshell ash (CSA) and lime modification. The silica fume-stabilized soil (SFSS), initially characterized by high plasticity, exists in a dispersed state. However, with the augmented absorption of ions due to the CSA and lime modification, the thickness of the double layer surrounding the SFSS molecules reduces, reducing repulsive forces between them. This phenomenon led to the formation of aggregated conglomeration in SFSS molecules. The formation of aggregated conglomerates enhances resistance against capillary uptake, impacting volumetric shrinkage and increasing the shrinkage void ratio as moisture content decreases. Consequently, the silica fume-stabilized soil (SFSS) composition stabilized with clamshell ash (CSA), and lime has notably endured within the aggregation domain.

Based on the aforementioned findings, the highest proportion of CSA and lime within the soil-stabilizing system (SFSS) led to the formation of a highly porous structure capable of retaining a significant volume of water around the soil molecules^47,48^, thus increasing the LL, PL, FSI (Free Swell Index), and SL value. The incorporation of CSA and lime into the stabilized soil-stabilizing system (SFSS) results in a structurally robust framework that effectively resists contraction, thereby contributing to an increase in the shrinkage limit (SL)^49^. Presently, the SFSS predominantly consists of non-swelling soil with reduced fine content, and the influence of the dispersed double-layer recession owing to the minimal presence of CSA and lime is negligible. Nevertheless, the increment percentages of CSA and lime in the SFSS led to a significant increment in PL and LL, while a slight marginal improvement in FSI and SL was ascribed to the emergence of calcium silicate hydrate (CSH) and calcium aluminate silicate hydrate (CASH) gel that withstands the large quantity of water. Hence, it can be inferred that the integration of CSA and lime into SFSS at multiple mix ratios can alter the plasticity and swell-shrinkage properties of the soil, and the integration of CSA into the SFSS shows a higher alteration compared to the integration of lime into the SFSS. The shrink-swell characteristics results obtained in this study are aligned with an assessment performed by Gadouri^14^. The utilization of pozzolanic materials can significantly modify the shrink-swell characteristics of the clayey soils.

### Effect of CSA and lime on the compaction properties of SFSS

**Figure 6:**
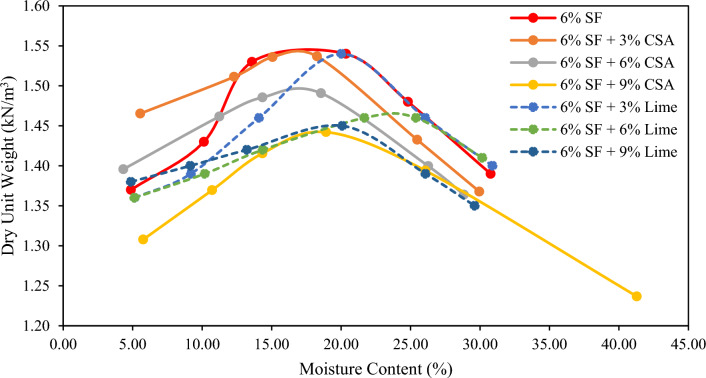
Variation of dry unit weight versus moisture content in SFSS admixed with CSA and lime.

The dry unit weight and moisture content of SFSS with CSA and lime content are illustrated in Fig. 6. The MDD value of SFSS is 1.54 g/cm^3^ with an OMC of 17.00%. The reduction of MDD is examined to be in the range of 1.42 g/cm^3^ to 1.55 g/cm^3^ with an OMC value ranging from 17.00 to 24.00%. Hasan et al.^50–52^ have reported a similar result. It is examined that when the dosage of soil stabilizers increased, the MDD decreased while the OMC values increased. The upsurge in OMC was proportionately confined. As a result, the reduction in thickness of the double-layer ensued, leading to grain accumulation as sodium cations in the diffuse layer of SFSS are replaced by calcium ions^53^. The prevalence of silty soil type within the SFSS matrix promotes closely packed molecules, thereby inhibiting the formation of additional void volume. Coherently, as the attraction between soil molecules increases, the molecules move closer together, reducing MDD value. The MDD reduction observed in both SFSS combined with CSA, and lime can be attributed to hydration, dissociation, and pozzolanic reactions, which decrease the density of the soil matrix. The addition of CSA and lime altered the organization of SFSS molecules, resulting in a denser packing arrangement. This alteration in specific surface area necessitates water absorption to facilitate hydration and pozzolanic reactions among the fine particles, thereby initiating soil improvement. The reduction in MDD is directly dependent on the OMC. Consequently, the OMC of the stabilized sample experiences flocculation due to the adhesion capability of CSA and lime. A substantial amount of water is required to form calcium silicate hydrate (CSH) and calcium aluminate hydrate (CAH) molecules and for the pozzolanic response in the presence of SF. The compaction results obtained in this study are in a good agreement with a study conducted by Gadouri^14^. The utilization of additives can significantly alter the compaction characteristics of the soil.

### Effect of CSA and lime on the UCS of SFSS

Improvement of UCS for 6% of SFSS altered with CSA and lime is portrayed in Table 6. The findings show that the compressive strength of the SFSS elevates slowly with the increment of the CSA and lime content and curing days. The compressive strength of the SFSS altered with 3%, 6%, and 9% of CSA and lime significantly improved from 15.51 kN/m^2^ at 1 day of curing to 93.54 kN/m^2^, 145.34 kN/m^2^ and 189.75 kN/m^2^ at 30 days of curing for CSA utilization and 27.17 kN/m^2^, 40.22 kN/m^2^, and 70.24 kN/m^2^ at 30 days of curing for lime utilization. From the results, the utilization of 9% of CSA and 9% of lime at 30 days of curing periods led to the most significant enhancement in the UCS value of the SFSS. Identical observations were observed by Alrubaye et al.^19^, Zaini et al.^54^, and Türköz et al.^55^. Besides, it is noted that the enhancement of the UCS observed in SFSS soil is due to the loess matrix that potentially fills the macropores and enhances the cohesion between loess aggregates. Additionally, the SF alters the sample's inner structure, resulting in an increment in the shear strength. The chemical effects stem from reactive silica in the SF, capable of chemically reacting with the loess particles in an alkaline surrounding, thereby producing a binding material^56^. This chemical reaction is also positively correlated with the observed improvement in sample strength. The massive CaO content present in CSA and lime is crucial to initiate the hydration process for the generation of Ca(OH)_2_ when reacting with water. The higher the Ca(OH)_2_ production, the higher the pozzolanic response will occur. High production of CSH and CASH leads to the effective alterations and substantial strength improvement of kaolinite clay. However, the significant physiochemical responses governing the engineering characteristics of SFSS mixtures include cation exchange reactions, flocculation of kaolinite clay particles, pozzolanic response, and agglomeration. The agglomeration of soil molecules resulted from the charge neutralization effect of the CSA and lime, forming silica gel. This phenomenon increased shear resistance and particle contact within the soil. Furthermore, reactions such as cation dissociation and interchange contribute to rapid changes in soil workability, enhancing particle adhesion and improving the kaolinite clay's strength. The UCS results obtained in this study are in a good agreement with a study conducted by Aldaood et al. ^11^. The utilization of additives can significantly alter the UCS value of the clayey soil.

**Table 6:** UCS of 6% SFSS modified with CSA and lime at various curing days.

Type of sample	Unconfined compressive strength with respect to curing days (kN/m^2^)			
	1 Day	7 Days	14 Days	30 Days
SFSS	15.51	21.84	23.06	24.53
SFSS + 3% CSA	26.81	65.93	69.16	93.54
SFSS + 6% CSA	27.16	79.83	127.92	145.34
SFSS + 9% CSA	33.26	85.41	166.50	189.75
SFSS + 3% Lime	21.33	25.14	26.49	27.17
SFSS + 6% Lime	26.70	27.10	29.35	40.22
SFSS + 9% Lime	27.38	43.08	50.32	70.24

In addition, the curing periods affect the inclusion of CSA and lime into the soil together with SF in the kaolinite clay stabilization process. As examined in Table 6, the ramification is notable at later curing periods. The increments in the specimens' curing periods resulted in the maximum value of UCS observed in SFSS altered with CSA and lime. Table 6 illustrates the influence of curing periods on the improvement of strength of the SFSS altered with CSA and lime. Based on the analysis, it can be clearly seen that the curing periods affect CSA and lime alteration on the SF-kaolinite clay stabilization. The CSA and lime-modified strength curves are significant compared to the strength curves of pure SFSS. The data depicted in the figure suggests that incorporating clamshell ash (CSA) and lime into the silica fume-stabilized soil (SFSS) has substantial positive ramifications on enhancing the long-term strength of the kaolinite clay soil. Additionally, it is evident from Table 6 that variations in curing periods notably influence the strength enhancement of CSA and lime-modified SFSS.

**Figure 7:**
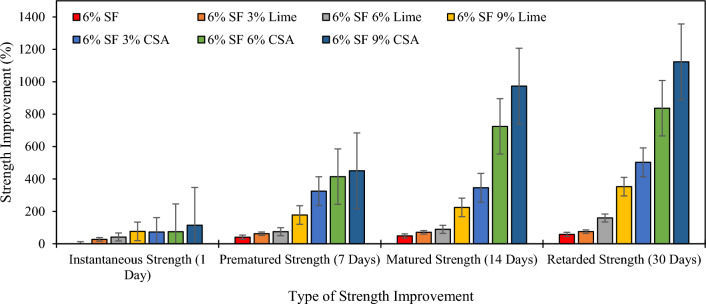
Percentage strength improvement of SFSS with CSA and lime content at different curing periods.

In this study, the strength improvement in percentage was also analyzed to portray the significant contribution of CSA and lime in improving the strength of the soil. Figure 7 illustrates the percentage strength improvement of CSA and lime content utilized to alter the SF stabilization activity. The analysis examined the effects of CSA and lime on strength improvement across four (4) contrasting curing periods. The strength improvement at 1 day of curing was represented as “instantaneous strength,” 7 days of curing as “premature strength,” 14 days of curing as “matured strength,” and 30 days of curing as “retarded strength.” The original strength of SFSS was established as a control for evaluating the percentage of strength improvement owing to the inclusion of CSA and lime for 1, 7, 14, and 30 days of curing periods, which disclosed the extent of the strength improvement coherent to pure SFSS. CSA and lime integration into the SFSS emerged with the lowest instantaneous strength improvement of 72.83% with 3% ESA and 27.27% with 3% lime, while 6% SFSS altered with 9% CSA and 9% lime resulted in a substantial instantaneous strength improvement of 114.41% and 76.48% respectively. The increment in the curing periods resulted in a significant increase in premature strength, matured strength, and retarded strength observed in 9% of CSA and lime utilization with a maximum percentage of strength improvement of 450.61%, 177.71% for premature strength improvement, 973.36% and 224.42% for matured strength improvement, and 1123.25% and 352.82% for retarded strength improvement. Therefore, it is perspicuous that the inclusion of CSA and lime into the SFSS emerged in a 72%-115% of instantaneous strength improvement of CSA, 27%-77% of instantaneous strength improvement of lime, 325%-451% of premature strength improvement of CSA, 62%-178% of premature strength improvement of lime, 345%-974% of matured strength improvement of CSA, 70%-225% of matured strength improvement of lime, 503%-1124% of retarded strength improvement of CSA, and 75%-353% of retarded strength improvement of lime. The results obtained are consistent with the findings reported by Zaini and Hasan ^26,40^.

### Effect of CSA and lime on the shear strength characteristics of SFSS

**Figure 8:**
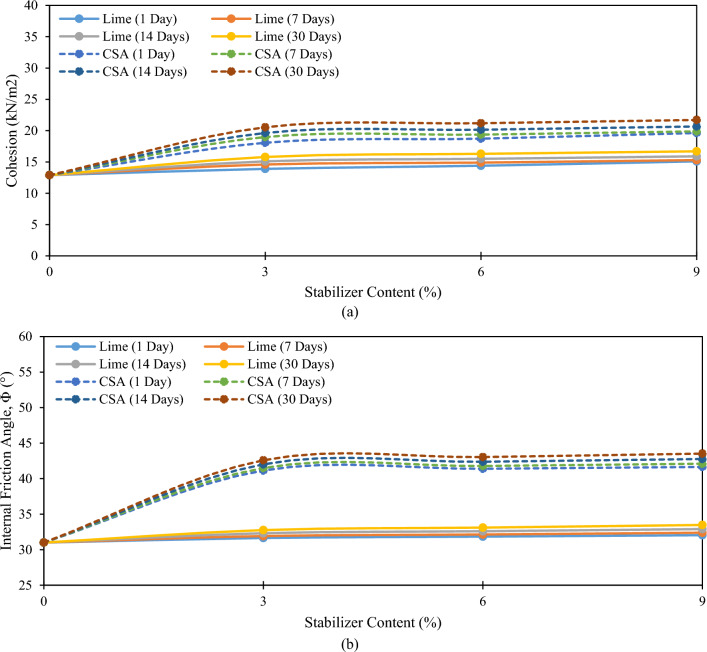
Effect of CSA and lime on the (a) Cohesion, c (kN/m^2^), and (b) Internal Friction Angle, Φ (°) of 6% SFSS.

The experimental results of the CIU analysis propose that the cohesion and internal friction angle of SFSS are significantly affected by the inclusion of CSA and lime (see Fig. 8a,b). The cohesion of SFSS, when CSA and lime were integrated exhibits improvement and reaches its maximum value of 19.63 kN/m^2^ and 15.10 kN/m^2^ at 1 day of curing periods when 9% CSA and lime were utilized respectively. The cohesion value of SFSS steadily increased with the increment in curing periods and achieved its highest value of 21.71 kN/m^2^ and 16.70 kN/m^2^ at 30 days of curing. The increase in the cohesion value of the SFSS was due to the reaction of cementitious material via hydration and the pozzolanic response process. The cohesion between SFSS molecules and the CSA and lime molecules increases through the chemical reaction. Furthermore, the small molecules of the soil stabilizers contribute to the packing of the soil matrix, emerging in a denser soil mixture. This densification is ascribed to the pozzolanic response, ultimately increasing the soil cohesion^37^. Comparable investigations have been assessed by Alrubaye et al.^18,19^ and Zaini and Hasan^46^.

Figure 8b highlights the influence of CSA and lime on the internal friction angle of SFSS. Based on the figure, the inclusion of CSA and lime at different proportions and curing periods increases the internal friction angle of the SFSS. The curing periods are essential in enhancing the internal friction angle of the SFSS. The increment of the internal friction angle value with an increasing percentage of CSA and lime in SFSS up to a maximum value of 43.52° and 33.48° at 30 days of curing can be elucidated by the collaborative impact of CSA and lime on the soil's mechanical characteristics. The maximum internal friction angle observed in all samples stabilized for 30 days of curing can be ascribed to the cation exchange activity and pozzolanic response between the SFSS-CSA and SFSS-lime. CSA and lime contain high calcium carbonate content and engage in a pozzolanic reaction with SiO_2_ contained in SFSS during stabilization. This chemical interaction gives rise to the formation of CASH and CSH. As the concentration of these compounds rises with higher proportions of CSA and lime, the cementation between soil particles is intensified. Throughout this process, the bonding between the particles becoming stronger, coupled with microstructural changes induced by the additives, fosters a more stable and interconnected soil matrix. The fine particles in CSA and lime further contribute to improved particle packing, filling void spaces, and enhancing the interlocking of soil grains. The cumulative effect of these mechanisms results in increased soil strength, translating into a higher internal friction angle. This phenomenon signifies an augmented resistance to shear and improved frictional characteristics within the stabilized soil, highlighting the effectiveness of CSA and lime in fortifying the engineering characteristics of the soil matrix. The cohesion and internal friction angle results obtained in this study are aligned with a study conducted by Harichane et al.^12^. The utilization of additives can significantly improve the shear strength parameters of the soil.

### Effect of CSA and lime on the mineralogical and morphological microstructure characteristics of SFSS

**Figure 9:**
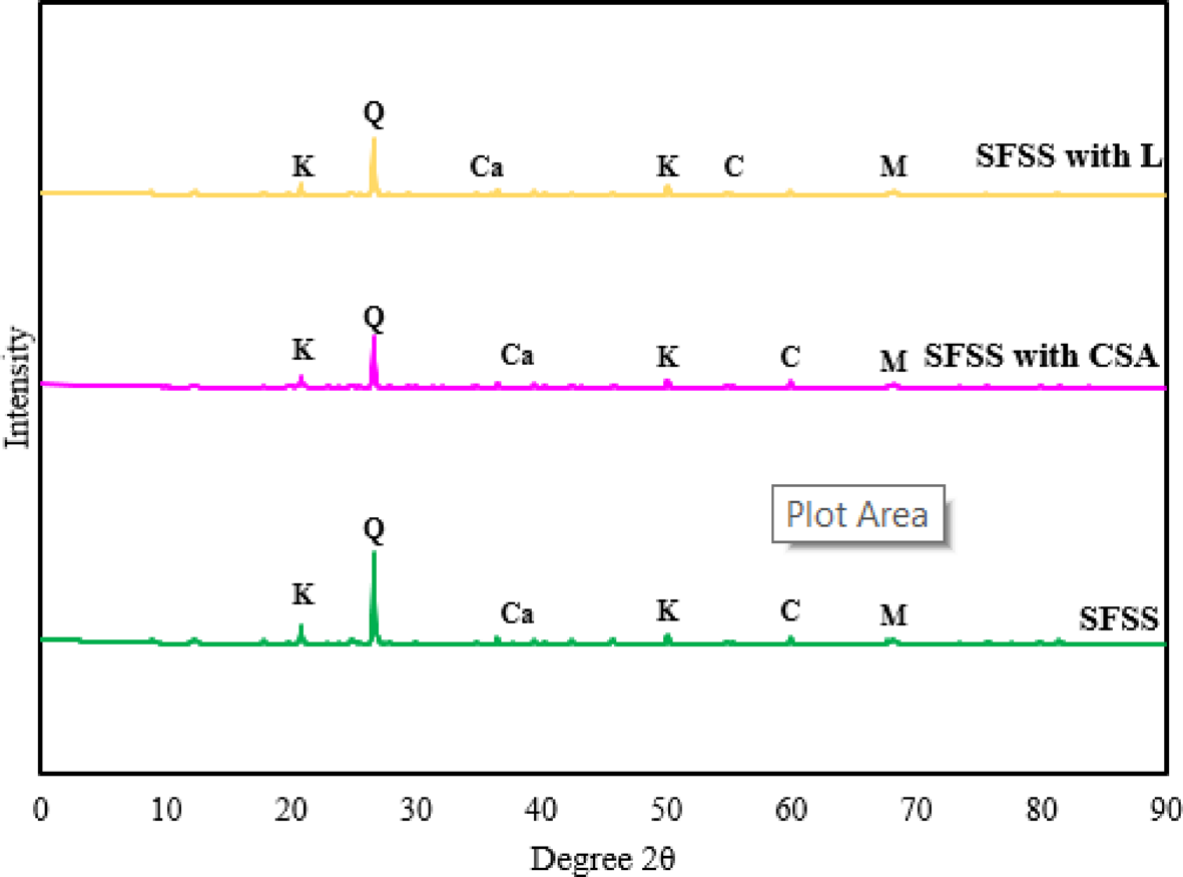
XRD pattern of SFSS with CSA and lime inclusion.

The notable strengthening effects observed during lime stabilization can be attributed to establishing pozzolanic products, such as CSH and CAH minerals^43,57^. X-ray diffraction (XRD) analysis provided insights into the mineralogical changes in the soil-stabilizing system (SFSS) upon the addition of clamshell ash (CSA), and lime, as illustrated in Fig. 9. The predominant minerals in the SFSS included quartz, kaolinite, illite, and cristobalite, while the introduction of CSA introduced calcite and portlandite, and lime added calcite. The CSA and lime-altered SFSS exhibited a composition of kaolinite, quartz, calcite, cristobalite, and muscovite. XRD patterns revealed new peaks, portraying the emergence of mineralogical compounds based on the added materials. The depletion in peak intensities of quartz and kaolinite in the stabilized kaolinite clay can be ascribed to the development of cementitious bonding. Previous studies conducted by Zaini and Hasan^40^ have examined the behaviour of silica fume (SF), eggshell ash (ESA), and lime stabilizers in kaolinite clay. Zaini and Hasan^40^ identified recurring crystalline clay minerals, including kaolinite, illite, smectite, and montmorillonite. The presence of calcium oxide (CaO) in the calcite and portlandite minerals of CSA and lime augmented the strength potential of the soil-stabilizing system (SFSS). Upon hydration, CaO produces calcium hydroxide (Ca(OH)_2_), which undergoes carbonation upon interaction with CO_2_ in the environment, causing the emergence of calcium carbonate (CaCO_3_). This carbonation mechanism enhances the bonding between soil molecules, contributing significantly to the strength of the SFSS and incorporating calcite and portlandite with wet clay particles in the presence of cristobalite results in the dissociation of calcium, silicate, aluminate, and hydroxide ions. The positive charges of calcium ions are absorbed by the negative charges of the SFSS, causing SFSS molecules to aggregate and ultimately enhancing cohesion among SFSS molecules, thereby increasing strength^58^. In line with this, it has been demonstrated that the inclusion of CSA and lime significantly alters pozzolanic responses, contributing to strength improvement. Consistent findings have also been documented by Alrubaye et al.^18^, Alrubaye et al.^19^, and Aldaood et al.^11^.

**Figure 10:**
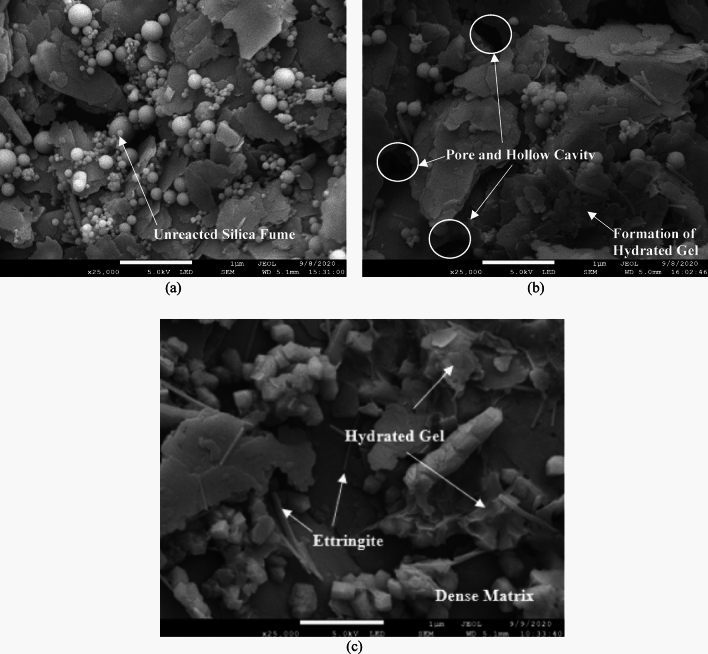
Morphological microstructure of (**a**) 6% SFSS; (**b**) 6% SFSS with 9% CSA; (**c**) 6% SFSS with 9% of lime.

Morphological microstructure analysis was conducted to assess the microstructural development in the matrix of SFSS (Fig. 10). The kaolinite clay displayed an uneven-shaped microstructure, while SF molecules exhibited a tiny spherical shape with a particle size of a thousand micrometres. CSA and lime particles had a bumpy, dispersed morphology with varying particle size distribution. The addition of CSA and lime resulted in morphological alterations, breaking the scabrous structure of SFSS into a lumpy structure. Needle-like crystals formed between the soil and cementitious molecules, creating a densely packed and stiffer soil structure. In the SFSS, SF particles showed limited reactivity and binding with the kaolinite clay molecules due to lower Ca(OH)_2_ production during the hydration process.

In contrast, 6% SFSS with 9% of CSA and lime exhibited a dense matrix with cementitious compound formations. The appearance of unpigmented cementitious compounds on the surfaces of soil-stabilizing system (SFSS) particles signifies the onset of pozzolanic reactivity. The microstructural development showed aggregated arrangements due to flocculation, where Ca^2+^ ions provided by CSA and lime reacted with alumina and silica in SFSS during the pozzolanic response. The integration of CSA and lime as additives in SFSS stabilization led to the flocculation of soil particles, reducing pore spaces and substantially increasing the strength of the SFSS. The morphology and size of ettringite crystals notably impacted the strength characteristics, with larger crystals demonstrating higher strength properties. Consequently, it can be inferred that the morphological microstructure of the altered soil aligns with the observed strength outcomes. In accordance with this, the amalgamation of SFSS, clamshell ash (CSA), and lime at 6% and 9% effectively enhanced soil strength by fostering denser particle arrangements.

### Stabilization mechanism of CSA and lime in SFSS Stabilization

Cation exchange (CEC), flocculation, and pozzolanic reaction are necessary for stabilizing problematic and expansive soil. CEC and flocculation are short-term reactions triggered immediately when the chemical stabilizing agent is added to the untreated virgin soil. CEC and flocculation are the critical reactions that cause the alteration of plasticity characteristics of soil. The resulting soil will become friable, whereas the natural soil can be easily cut and moulded into shape when wetted. The reducing power of CEC is influenced by the adsorption affinity of cation, whereas Al > Ca > Mg > K = NH > Na > Li, pH of soil water, and types of clay minerals. Generally, kaolinite has the lowest CEC strength, while montmorillonite has the highest. Soil with high CEC value generally exhibits greater swelling properties than soil with lower CEC^59–61^.

According to Huang et al.^62^, the concentration of calcium ion (Ca^2+^) and OH^-^ from saturated Ca(OH)_2_ affects the production of calcium silicate hydrate (CSH), with chemical formula CaSiO_3_H_2_O in the stabilized soil, and CSH is the main contributor to the strength of stabilized soil. When the concentration of Ca^2+^ and OH^-^ is low, the strength of stabilized soil is low due to the lack of CSH. Yu et al.^63^ also deduced that CEC can decrease the saturation of Ca(OH)_2_, which increases the concentration of Ca^2+^ and OH^-^ and the production of CSH; thus, the strength of the stabilized soil improved. If the CEC of the soil sample is too high, Ca^2+^ is exchanged by the other cations adsorbed on the soil particles, thus reducing the Ca^2+^ concentration, and the stabilized soil strength is reduced due to insufficient CSH.

Flocculation is when the dispersed particles form larger clusters, come into contact, and adhere together. Generally, most soil particles are negatively charged, and due to the repelling charges theory, the soil particles are dispersed. When the stabilizer is introduced to the untreated clay soil, the cations are attracted by the negative charge of clay particles, causing them to stick together. Flocculation occurs as the repulsive force between the negative charge of clay particles is cancelled off when they are attracted by the stabilizer's Ca^2+^ or magnesium ion (Mg^2+^) and form a friable soil. Cementitious properties and pozzolanic reactions are crucial in stabilizing soft clay soil. The pozzolanic reaction, which is a slow process, primarily contributes to strength development. Typically, CaO and SiO_2_ are recognized as cementitious material's principal constituents, both acting as binding agents when water is added. When hydration occurs, when water is added, the CaO will react with the water and form calcium hydroxide, Ca(OH)_2_, as shown in Eq. (1).

1 $$CaO+{H}_{2}O\stackrel{Hydration}{\to } {Ca(OH)}_{2}$$

However, dissociation reactions lead to immediate changes in the plasticity and workability of the clay soil, as described in Eq. (2). Processes such as cation exchange, dispersion of clay soil particles, accumulation, and pozzolanic reactions are the primary physicochemical responses that affect the properties of SiO_2_-CaO soil mixtures. The presence of Ca^2+^ ions in the stabilized clay soil reacts with water according to the chemical reaction pathways outlined in Eqs. (3 and 4).

Dissociation Reaction:

2 $${Ca(OH)}_{2}\stackrel{a}{\to }{Ca}^{2+}+{2[OH]}^{-}$$

Pozzolanic reaction:

3 $${Ca}^{2+}+{2[OH]}^{-}+ {SiO}_{2}\stackrel{a}{\to }CSH$$

4 $${Ca}^{2+}+{2[OH]}^{-}+ {Al}_{2}{O}_{3}\stackrel{a}{\to }CASH$$

The continuous pozzolanic reaction produces gel-like calcium silicate hydrates (CSH) and threadlike calcium aluminate silicate hydrates (CASH), facilitating the bonding between stabilized clay soil particles and reinforcing soil strength. The prolonged pozzolanic reaction significantly influences the engineering properties of the soil, including porosity, hydraulic conductivity, and strength. Kaolinite clay has a low CEC due to its aluminosilicate composition, whereas CSA and SF have greater CEC values. The greater CEC of SFSS with CSA allows an adsorbtion of calcium (Ca^2+^) and aluminum (Al^3+^) ions from the surrounding solution, encouraging the production of CASH gel by the interaction of these ions with the clay's silicate components. The CASH gel functions as a binding agent, stabilizing the kaolinite clay while increasing its strength and durability. As a result, the CEC of SFSS with CSA is critical in promoting the development of CASH gel and improving the stability of stabilized kaolinite clay. According to Shao et al. ^64^ and Barman and Dash ^5^, the pozzolanic reaction depends on two key factors: the maximum amount of Ca(OH)_2_ that the pozzolan can react with and the surface area of the pozzolan. A larger surface area of the pozzolan results in higher pozzolanic reactivity. Clamshell ash (CSA) and lime primarily contain CaO, while silica fume (SF) consists of over 80% SiO_2_ and is considered an artificial pozzolan primarily composed of silica glasses^65^. As pozzolans typically have low CaO content, lime or cement is often added due to their high CaO percentage. In this study, CSA and lime are utilized as the CaO source to replace cement in the chemical stabilization of soil.

### Cost analysis

**Figure 11:**
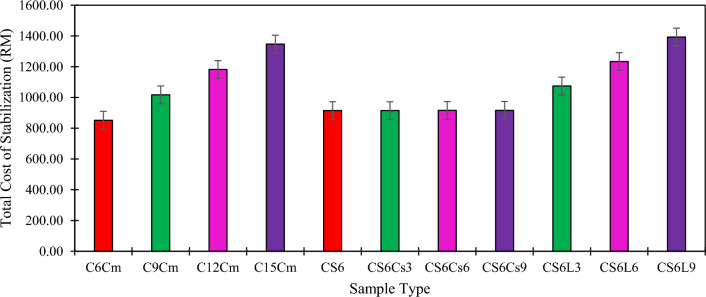
Cost analysis comparison between cement and soil stabilizers utilized in the study.

In the quest for cost-effective strategies in civil engineering substructure development, navigating within the constraints of limited funding allocated for civil infrastructure construction and maintenance is imperative. To assess the efficacy of the proposed soil stabilization materials introduced in this study compared to conventional cement-based soil treatment, a cost–benefit analysis emerges as a pivotal parameter for decision-making amid many alternatives. Accordingly, the pertinent literature was surveyed to gather precise unit prices of commodities, utilities, excavation, and in-place stabilization. Operational costs beyond these parameters were excluded from consideration. Table 7 presents the unit prices of materials and operations for soil stabilization. Leveraging the insights gleaned from this table, the soil stabilization process of the soil-stabilizing system (SFSS) was scrutinized and depicted in Fig. 11.

**Table 7:** The unit price of materials and operations for soil stabilization.

Materials and process	Unit price (RM/kg)	Source of data
Clayey Soil	0.00	^66^
Cement	0.55	^67^
Silica Fume	0.65	^31^
Clamshell Ash	0.00	N/A
Hydrated Lime	0.53	Ratnadeep Chemicals Supplier
In-place Stabilization	0.02	^68^
Water	2.86*	Suruhanjaya Perkhidmatan Air Negara (SPAN)
Excavation	0.032	Jabatan Kerja Raya (JKR) Malaysia

*The rate was referred to Selangor State rate = RM2.86 / m^3^.

Table 7 shows that the unit price for cement as soil stabilizer is lower than SF, with a margin difference of RM0.47 per kg (C-SF). Due to the data source found on the CSA and the material was self-collected, the CSA price was considered RM0. Hence, compared to CSA, cement's unit price is slightly higher, with a margin difference of RM0.55. The unit price of materials between cement and hydrated lime are RM0.55 and 0.53, respectively. The unit price shown in Table 7 is crucial to determine the total cost required to stabilize the kaolinitic clay soil using ideal percentages and combinations of SF, CSA, and lime. Based on the proposed percentages, the quantity of stabilizers needed to stabilize the kaolinitic clay soil must be calculated to calculate the total cost required for soil stabilization. Hence, Table 8 shows the quantity of soil stabilizers needed to stabilize the assumed quantity of soil. Based on Table 8, the total soil assumed to be stabilized in this study was equal to 10,000 kg (10 m^3^), as discussed by Tripathi et al. ^68^.

**Table 8:** Quantity of soil stabilizers needed to stabilize the assumed quantity of soil.

Type of sample	Percentage of stabilizer (%)				Quantity of stabilizer (kg)			
	Cm	S	Cs	Lime	Cm	SF	CSA	Lime
C6Cm	6	0	0	0	600	-	-	-
C9Cm	9	0	0	0	900	-	-	-
C12Cm	12	0	0	0	1200	-	-	-
C15Cm	15	0	0	0	1500	-	-	-
CS6	0	6	0	0	-	600	-	-
CS6Cs3	0	6	3	0	-	600	300	-
CS6Cs6	0	6	6	0	-	600	600	-
CS6Cs9	0	6	9	0	-	600	900	-
CS6L3	0	6	0	3	-	600	-	300
CS6L6	0	6	0	6	-	600	-	600
CS6L9	0	6	0	9	-	600	-	900

Cm, Cement; C, Kaolinite Clay; S, Silica Fume; Cs, Clamshell Ash; L, Lime; Quantity of Soil based on 10 m^3^ Volume = 10 000 kg.

Based on the total soil needed to be stabilized, the quantity of soil stabilizers was calculated and tabulated in Table 8. In order to compare the cost between the traditional stabilizers and the proposed stabilizers, four (4) samples that utilized cement stabilizers were tallied with the seven (7) types of proposed stabilizers. C6Cm sample was compared with the CS6 sample, the C9Cm sample was compared with the CS6Cs3 and CS6L3 sample, the C15Cm sample was compared with the CS6Cs6 and CS6L6 sample, and the C15Cm sample was compared with the CS6Cs9 and CS6L9 sample. In this study, 10 m^3^ of soil is assumed to be stabilized. Coherent to the unit price of materials and operations for soil stabilization, the total cost projected for the conventional and proposed soil stabilizers is shown in Table 9. Figure 11 compares the total cost between cement and the soil stabilizers used in the study. The OMC of the cement was selected to be 7% based on the research conducted by Kulanthaivel et al. ^69^.

**Table 9:** Cost analysis of projected soil to be stabilized using conventional soil stabilizers and proposed soil stabilizers.

Type of sample	Quantity of stabilizer (kg)				Total cost (RM)
	Cm	S	Cs	Lime	
C6Cm	600	-	-	-	852.12
C9Cm	900	-	-	-	1017.18
C12Cm	1200	-	-	-	1182.24
C15Cm	1500	-	-	-	1347.30
CS6	-	600	-	-	915.15
CS6Cs3	-	600	300	-	914.83
CS6Cs6	-	600	600	-	916.09
CS6Cs9	-	600	900	-	916.25
CS6L3	-	600	-	300	1074.92
CS6L6	-	600	-	600	1234.09
CS6L9	-	600	-	900	1393.25

Cm, Cement; C, Kaolinite Clay; S, Silica Fume; E, Eggshell Ash; L; Lime; Total Cost = (Stabilizer Quantity x Unit Price of Materials) + (In-place Stabilization x Soil Quantity) + (Water Rate x (OMC x Stabilizer Quantity) + Soil Quantity) + (Excavation x Soil Quantity).

Based on Table 9 and Fig. 11, the comparison is made between C6Cm with CS6 (denoted with red colour), C9Cm with CS6Cs3 and CS6L3 (denoted with green colour), C12Cm with CS6Cs6 and CS6L6 (denoted with pink colour), and C15Cm with CS6Cs9 and CS6L9 (denoted with purple colour). The table and figure show that the total cost of stabilization for cement utilization as a soil stabilizer is cheaper than SF stabilizer, with a 6.96% cost increment (margin cost difference of RM63.03). Meanwhile, the utilization of CSA with SFSS is cheaper than cement, with a margin cost difference of RM102.03, RM267.41, and RM431.22, respectively. Conversely, the utilization of lime with SFSS resulted in higher increment of the total cost of stabilization lime utilization when compared to cement, with an increment cost of RM57.74, RM51.84 and RM45.95, respectively. Therefore, it can be concluded that utilizing CSA in SFSS can reduce the total cost of soil stabilization up to 10.06% (with 3% CSA utilization), 22.51% (with 6% CSA utilization), and 31.99% (with 9% of CSA utilization), while utilizing lime in SFSS can increase the total cost of stabilization up to 5.37% (with 3% lime utilization), 4.20% (with 6% lime utilization), and 3.30% (with 9% of lime utilization) compared to the utilization of cement as soil stabilizing agent. Nevertheless, due to the cost-effectiveness of CSA utilization in SFSS, this material is an ideal choice for stabilizing problematic and expansive soil^70-72^.

## Conclusions

Various laboratory tests were performed to examine the effect of using CSA and lime on SFSS at various percentages and curing periods. The examination involved investigating the strength, plasticity, shrink-swell behaviours, compaction characteristics, and mineralogical and microstructural characteristics of kaolinite clay soil stabilized at OSFC and altered using CSA, an agricultural waste collected from the food service industry and lime. Therefore, the conclusions can be drawn as follows:

- Adding clamshell ash (CSA) and lime reduces the plasticity of the stabilized soil with increasing dosages. However, CSA and lime modification significantly impacted the shrink-swell behaviour of the silica fume stabilized soil (SFSS). The results regarding plasticity and shrink-swell behaviour indicate that when sufficient silica fume (SF) content is present, CSA and lime modification significantly alter the behaviour of the stabilized soil.
- Alteration of OSFC-stabilized soil using CSA and lime emerged in the improvement of the strength of the modified soil. Curing periods significantly affect the strength of the SFSS altered with CSA and lime. The maximum strength benefit is accomplished at 30 days of curing with an enormous increase of 114.41% and 76.48% in instantaneous strength, 450.61% and 177.71% in premature strength, 973.36% and 224.42% in matured strength, and 1123.25% and 352.82% in retarded strength. Hence, CSA and lime alterations can substantially improve the strength of the OSFC-stabilized soil, and the maximum enhancement is seen in retarded strengths.
- The CIU test conducted on SFSS and SFSS altered with CSA and L shows noticeable cohesion and internal friction angle value alterations. The utilization of CSA and lime at 3%, 6%, and 9% cured at 1, 7, 14 and 30 days shows a significant increase in cohesion value and internal friction angle, with the maximum value recorded when 9% of CSA and lime were utilized and cured at 30 days of curing.
- The X-ray diffraction (XRD) analysis reveals a notable prevalence of matrix disturbance, accompanied by the formation of cementitious compounds such as calcium silicate hydrate (CSH), calcium aluminate hydrate (CAH), and ettringite. This signifies a progressive improvement in strength over time. The enhanced strength is attributed to developing a coating around the soil particles, in addition to flocculation. Field emission scanning electron microscope (FESEM) images of the silica fume stabilized soil (SFSS) altered with clamshell ash (CSA) and lime depict the evolution of a dense matrix with cementitious compounds formed within the soil matrix.
- The SFSS altered with 3%-9% CSA as the environment-friendly and cost-effective method can reduce 10%-40% of the total cost of stabilization while utilizing 3%-9% of lime resulted in an increment of the total cost of stabilization around 3%-5% compared to stabilizing the soil with cement.
- Consequently, the study concludes that the incorporation of clamshell ash (CSA) and lime significantly influenced the engineering properties and enhanced the strength of the silica fume stabilized soil (SFSS). The high content of calcium oxide, silicon dioxide, and aluminium oxide in the SFSS modified with CSA and lime contributed to the most substantial alterations and the maximum improvement in engineering properties and shear strength of the SFSS.
- The improvement in strength and densification of stabilized soil matrix through the formation of cementitious phases and pozzolanic responses involves several key mechanisms. When lime and clamshell ash are admixed with the soil with a presence of water, a reaction occurred to form calcium silicate hydrates (CSH) and calcium aluminate silicate hydrate (CASH). These compounds act as binders, creating bridges between soil molecules and encouraging cohesion within the soil matrix. Additionally, pozzolanic reactions occur between the silica fume and lime, further enhancing the generation of CSH and filling voids within the soil structure. This process increases the interlocking of soil particles, reducing void spaces and enhancing the overall compactness and strength of the soil. Moreover, the pozzolanic responses contribute to the long-term durability of the stabilized soil by reducing hydraulic conductivity and increasing resistance to chemical attack and environmental degradation. Overall, the effects of cementitious phase formation and pozzolanic responses lead to substantial improvements in the mechanical properties and stability of the stabilized soil matrix, making it more suitable for various engineering applications.
- This research has important applicability in real-world engineering projects and can help with numerous elements of infrastructure development and environmental sustainability. Optimized combinations of silica fume, clamshell ash, and lime can be utilized to increase the strength and durability of road pavements, particularly in locations with expanding clay soils. Engineers can apply the findings to road construction projects by using stabilized soil mixes as sub-base or base materials, lowering pavement failure rates and maintenance costs.

## Data Availability

The datasets generated during and/or analysed during the current study are available from the corresponding author on reasonable request.
