# Author Correction: Experimental investigations on physico-mechanical properties of kaolinite clay soil stabilized at optimum silica fume content using clamshell ash and lime

**DOI:** 10.1038/s41598-024-64128-y

**Published:** 2024-06-10

**Authors:** Muhammad Syamsul Imran Zaini, Muzamir Hasan, Sultan Almuaythir, Masayuki Hyodo

**Affiliations:** 1 Faculty of Civil Engineering Technology, Universiti Malaysia Pahang Al-Sultan Abdullah, Lebuh Persiaran Tun Khalil Yaakob, 26300 Kuantan, Pahang Malaysia; 2 Department of Civil Engineering, College of Engineering in Al-Kharj, Prince Sattam bin Abdulaziz University, 11942 Al-Kharj, Saudi Arabia; 3 Graduate School of Science and Technology for Innovation, Yamaguchi University, Ube, Japan

Correction to: *Scientific Reports* 10.1038/s41598-024-61854-1, published online 14 May 2024

The Acknowledgements section in the original version of this Article contained an error.

“The authors express their appreciation to Prince Sattam bin Abdulaziz University for their generous financial assistance in supporting this research through project number PSAU/2024/R/1445."

now reads:

“This study is supported via funding from Prince Sattam bin Abdulaziz University project number PSAU/2024/R/1445.”

The original Article has been corrected.

